# Insights about MYC and Apoptosis in B-Lymphomagenesis: An Update from Murine Models

**DOI:** 10.3390/ijms21124265

**Published:** 2020-06-15

**Authors:** Eleonora Vecchio, Giuseppe Fiume, Serena Correnti, Salvatore Romano, Enrico Iaccino, Selena Mimmi, Domenico Maisano, Nancy Nisticò, Ileana Quinto

**Affiliations:** Department of Experimental and Clinical Medicine, University Magna Graecia of Catanzaro, 88100 Catanzaro, Italy; fiume@unicz.it (G.F.); serena.correnti95@gmail.com (S.C.); romano.salvatore396@gmail.com (S.R.); iaccino@unicz.it (E.I.); mimmi@unicz.it (S.M.); maisano@unicz.it (D.M.); nancynistico@unicz.it (N.N.)

**Keywords:** MYC, apoptosis, lymphomagenesis, mouse models, miRs, B-cells

## Abstract

The balance between cell survival and cell death represents an essential part of human tissue homeostasis, while altered apoptosis contributes to several pathologies and can affect the treatment efficacy. Impaired apoptosis is one of the main cancer hallmarks and some types of lymphomas harbor mutations that directly affect key regulators of cell death (such as BCL-2 family members). The development of novel techniques in the field of immunology and new animal models has greatly accelerated our understanding of oncogenic mechanisms in MYC-associated lymphomas. Mouse models are a powerful tool to reveal multiple genes implicated in the genesis of lymphoma and are extensively used to clarify the molecular mechanism of lymphoma, validating the gene function. Key features of MYC-induced apoptosis will be discussed here along with more recent studies on MYC direct and indirect interactors, including their cooperative action in lymphomagenesis. We review our current knowledge about the role of MYC-induced apoptosis in B-cell malignancies, discussing the transcriptional regulation network of MYC and regulatory feedback action of miRs during MYC-driven lymphomagenesis. More importantly, the finding of new modulators of apoptosis now enabling researchers to translate the discoveries that have been made in the laboratory into clinical practice to positively impact human health.

## 1. Role of MYC in Non-Transformed Cells

MYC is a family of transcription factors and consists of c-MYC, N-MYC, and L-MYC (in this review, MYC will refer to c-MYC unless otherwise specified) [1]. Through its direct and indirect activity, MYC regulates the expression of several genes involved in cell proliferation, differentiation, metabolism, cell growth, and apoptosis [2]. MYC works as a transcription factor through heterodimerization with its transcriptional co-factor MYC-associated protein X (MAX). MYC/MAX heterodimers bind the promoter of target genes through E-box motifs (CACGTG) to activate transcription [3]. The regulation of gene expression can involve multiple mechanisms including direct activation or inhibition of transcriptional machinery complex [4], induction of specific microRNAs [5], recruitment of chromatin remodeling complexes [6], mRNA stabilization and protein biogenesis [7]. The absence of MYC is not compatible with life [8] and its loss of function in mature cells arrests survival, proliferation, and cell development [9]. Thus, MYC is essential during both embryonic development and in adult mature cellular growth.

The expression of MYC protein is tightly regulated, at transcriptional, post-transcriptional and, post-translational level [10,11,12] in normal tissue, with a half-life of ~20 min [13].

The MYC protein is organized as follow: a N-terminal transactivation domain (TAD), MYC box domains (MB0-IV), a PEST domain (Proline, glutamic acid (E), Serine and Threonine rich), a nuclear localization sequence (NLS), and the carboxy-terminus basic-helix-loop-helix-leucine zipper (bHLHZ). MYC protein domains participate in binding to a huge set of partner proteins, which modulate MYC functions and contribute to regulating gene expression. Specifically, MB0-II motifs affect the activity and stability of MYC and mediate the association of MYC with several proteins including P-TEFb, FBW7, and PIN1. MBIII–IV motifs of MYC are pivotal for the roles of MYC in apoptosis and in its protein turnover. Another MYC key domain is represented by the bHLHZ motif that is required for the interaction between MYC and MAX, essential for DNA binding [14].

The *MYC* gene is activated by growth factors and is sensitive to nutrient availability. In vitro studies confirmed that serum stimulation of quiescent cells in culture induced a rapid up-regulation of MYC mRNA. Conversely, MYC expression is reduced by nutrient deprivation and hypoxia [10,15]. The tight regulation of MYC expression in non-transformed cells was remarkably responsive to extracellular signals, at both transcriptional and translational levels [16]. Because MYC is one of the most highly regulated proteins in the cell, many important regulatory mechanisms have been discovered through studies of MYC [17].

## 2. MYC and Lymphomagenesis

*MYC* is one of the most deregulated oncogenes in humans and is frequently overexpressed in hematological malignancies due to aberrations. In most cases, aberrations include gene amplification or translocation, leading to MYC overexpression and a change in protein function or protein conformation [18,19]. *MYC* rearrangements have a high frequency in the aggressive B-cell lymphomas [20]. In Burkitt’s lymphoma are present rearrangements in >90% of cases [21], in diffuse large B-cell lymphoma (DLBCL) about 5–14% of cases [22] and in Plasmablastic lymphoma (PBL) about 50% of cases [23]. *MYC* point mutations in the coding regions are another type of genetic aberration (e.g., in a majority of the Burkitt’s lymphomas). They can induce *MYC* oncogenetic deregulation and can be resumed as follow: the residues most frequently mutated are Thr58 and S62 in the MBI motif [24]; the F138C mutation in the MBII motif [25]; and deletion of residues 188–199 in the MBIII motif [26].

A role for MYC in driving human cancer was first identified in Burkitt’s lymphoma, where MYC deregulation was a result of chromosomal translocation into the immunoglobulin heavy chain locus [27]. *MYC* expression is deregulated and increased through different mechanisms in tumor cells. Several studies demonstrate unequivocally that the enhanced *MYC* expression is a common feature of tumorigenesis in both MYC-driven and non-MYC-driven tumors [28]. Recent studies suggest that lymphomagenesis in a pre-clinical mouse model *Eμ-myc* is also determined by MYC-cooperative mutations and concomitant multigenic lesions involving CDKN2a, NRAS, and KRAS, suggesting two-hit pathogenesis [29]. Combinations of MYC with other oncogenes including RAS [30], oncogenic EBV components [31], or MYC-surface CD19 signaling amplification loops have been shown to accelerate lymphoma development [32]. However, the suitability of these models to mirror therapeutic responses to target treatments has not been adequately tested. Notably, MYC promotes cell growth by stimulating the expression of the genes involved in the biogenesis of organelles such as ribosomes and mitochondria, glucose metabolism, and/or lipid synthesis. Furthermore, MYC is a pivotal player in the metabolic rewiring of cancer cells, the strategy that cancer cells adopt to cope with the energetic and metabolic demands required to support tumor growth and progression. [33,34,35]. MYC can contribute to tumorigenesis also influencing host immune cells [36]. Of note, MYC with STAT3 transcriptionally regulates PD-L1 in ALK-negative anaplastic large cell lymphoma (ALCL). In ALCL tumor-derived cell lines, MYC overexpression increased PD-L1 expression. Treatment with the BET inhibitor JQ1 or a MYC-targeting siRNA decreased PD-L1 expression [37]. In a panel of mouse MYC-driven lymphomas, tumor growth was dependent on MHC I expression that seemed to regulate innate immune surveillance [38,39].

## 3. MYC Expression as a Key Factor of Apoptosis Activation

MYC can promote cell activation, growth, and proliferation while concomitantly sensitizes cells to apoptosis. Normal cells have specific molecular sensors that enable detection of elevated MYC levels and response by triggering apoptosis. Conversely, transformed cells often acquire the ability to resist the apoptotic effects of elevated MYC and to respond, by increasing proliferation rates [17]. The abrogation of MYC-potentiated apoptosis is a crucial event of cellular transformation and cancer progression [40]. MYC can be conceived as a double face transcription factor since on one hand it supports cell proliferation and on the other apoptosis. Initially, it was considered disconcerting: a single molecule drives different functions. Quickly, these observations were confirmed for other oncogenes, including E2F1 and E1A [41]. Of note, premalignant cells allow an enhanced sensitivity to apoptosis caused by ectopic MYC expression, which they lose after malignant transformation [40]. Indeed, the tumor cells have acquired specific mechanisms for mitigating the apoptotic effects that MYC imposes in their normal counterparts. What follows are examples of key observations linking MYC to other apoptotic regulators.

## 4. Crosstalk between MYC and Cellular Regulators of Apoptosis in Lymphoma

**Figure 1:**
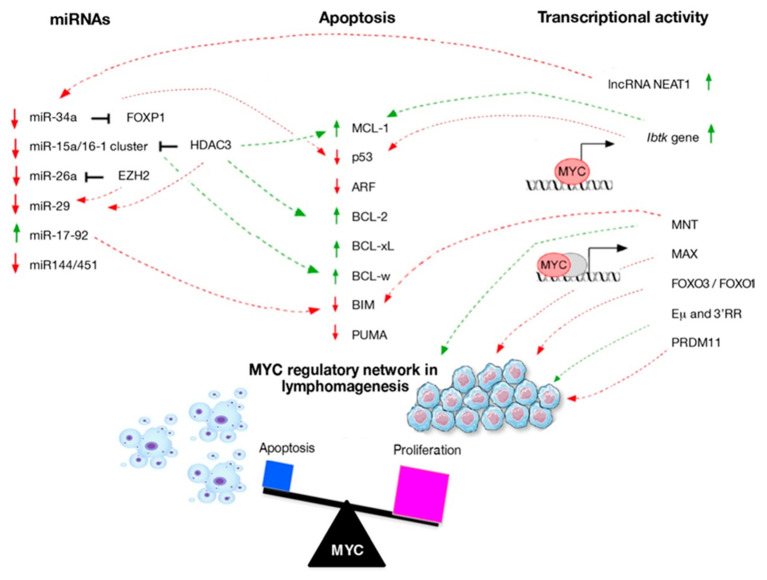
Regulatory networks involved in lymphomagenesis. Schematic representation of the molecular mechanism through which the initiating cancer B-cells escape from apoptosis. The interactors that are up- (arrow in green) and down-regulated (arrow in red) directly or indirectly by MYC are shown.

It is well known that MYC contributes to oncogenic changes and cell transformation but its aberration alone maybe not sufficient to initiate lymphomagenesis. Important advances have been made in elucidating how cells respond to MYC activation by enhancing proliferation or by triggering apoptosis. These mechanisms are interconnected and involve two main pathways: BCL-2 pathway, p53 pathway, or both. In the beginning, apoptosis can be regulated by three distinct subgroups of proteins belong to the same family: BCL-2 proteins. They are clustered as follow: the pro-survival proteins (such as BCL-2, BCL-xL, MCL-1, and BCL-w) are essential for cell survival; the BH3-only proteins (for example, BIM and PUMA) are crucial for apoptosis initiation; finally, BAX/BAK are required for apoptosis execution [42]. MYC sensitizes pre-cancerous cells to undergo apoptosis by altering the balance of pro- and anti-apoptotic factors (Figure 1). In the *Eμ-myc* model of lymphomagenesis, the Cleveland laboratory showed that MYC indirectly suppresses the anti-apoptotic proteins BCL2 and BCL-xL [43]. This is consistent with evidence showing that MYC triggers apoptosis through BAX. In this way, MYC activity directly influences cytochrome *c* release from the mitochondria, and therefore the activation of downstream effector caspases [42,44]. The *BCL-2* gene is the first cell death regulator identified by its frequent translocation in a well-studied subtype of lymphoma in humans, the follicular B-cell lymphoma (FL) [45]. Its oncogenic potential was revealed when BCL-2 overexpression was shown to promote the survival (but not proliferation) of pro-B-cells deprived of cytokines [46]. DLBCLs contain both rearrangements in BCL-2 and translocation of MYC. They are classified as “double-hit lymphomas” (DHLs) and represent ~10% of DLBCL cases [47]. Most recent discoveries have focused on the impact of two anti-apoptotic proteins of BCL-2 family, BCL-w and MCL-1 on MYC-driven lymphomagenesis. The overexpression of BCL-w was observed in DLBCL and correlated with poorer clinical outcomes. Of note, MYC can indirectly suppress BCL-w expression during lymphomagenesis, by up-regulating the miR-15 family members. Its loss profoundly delayed MYC-mediated B-cell lymphoma development due to increased B-cell apoptosis mediated by MYC. Loss of BCL-w sensitized B-cell to growth factor deprivation-induced B-cell apoptosis and suppressed MYC-induced lymphomagenesis, suggesting a crucial role of BCL-w in B-cell survival and lymphoma development [48]. MCL-1 protein attends in promoting the survival of B lymphoid progenitors that undergo MYC-driven lymphomagenesis. Loss of one *MCL-1* allele almost abrogated MYC-driven-lymphoma development owing to a reduction in lymphoma initiating pre-B-cells. [49]. Moreover, MCL-1 is critical also for the development of B-cells lymphoma and its down-expression in lymphomas can be compensated by altered levels of other cell death regulators, such as BIM and/or BCL-xL [50].

On the other hand, p53 is one of the key molecules involved in the pathogenesis of B-cell lymphomas. Lymphomas with co-existent MYC and p53 alterations/mutations are synergistic, resulting in more aggressive lymphomas, and patients have a particularly poor prognosis with short median survival time [51]. Previous studies demonstrated that the deregulation of MYC up-regulates ARF, which in turn activates p53 to regulate a pool of target genes involved in apoptosis and growth arrest [52]. ARF–MDM2–p53 pathway in MYC-induced apoptosis accelerated tumorigenesis through the loss of these tumor suppressors in mouse models of MYC oncogenesis [53,54,55,56,57,58,59]. Notably, *BMI-1* has been one of the first genes initially identified as an oncogene that cooperates with MYC in the generation of mouse pre-B-cell lymphomas by inhibiting INK4a/ARF proteins and by activating the human telomerase reverse transcriptase (h-TERT) [60].

One of the main p53 target genes is *PUMA*, a BH3-only pro-apoptotic protein. Previous studies observed that *puma* deletion in the *Eμ-myc* mouse accelerates MYC-driven lymphomagenesis [61]. Normally, PUMA initiates programmed cell death upon induction, triggering the cytochrome *c* release and caspase activation [62]. Conversely, deletion of *puma* efficaciously preserves cells against apoptosis induced by either DNA damage or oncogene activation, competing with the protection if p53 is deleted [61].

## 5. Transcriptional Regulation Network Involved in MYC-Driven Lymphomagenesis

Many other genes are involved in the pathogenesis of lymphoma cross-talking with *MYC*, mainly through transcriptional regulation (see in Table 1 and Figure 1). The first dimerization partner of MYC [63], MAX, have a role in MYC-induced oncogenesis. It is well to remember that overexpression of MAX alone in murine lymphoid cells is non-oncogenic and results in reduced B-cell proliferation. Noteworthy, B-cell-specific deletion of MAX has a modest effect on B-cell development but completely abrogates *Eμ-myc*-driven lymphomagenesis. Moreover, a study found that MAX loss leads to a significant reduction in MYC protein levels and down-regulation of direct transcriptional targets in premalignant *Eμ-myc* cells, including regulators of MYC stability [64]. Furthermore, the transcriptional repressor and MAX interactor MNT (MAX Network Transcriptional Repressor), competes with MYC for occupancy at E-box sequences in promoter regions [65]. Campbell and colleagues showed that *MNT* heterozygosity slowed MYC-driven tumorigenesis in *vavP-MYC10* and *Eμ-myc* mice, suggesting that MNT facilitates MYC-driven oncogenesis, although no differences in the number of preleukaemic cells were apparent [66]. Recently, Nguyen and colleagues clearer explained the phenomenon. They showed that MNT supports B-lymphomagenesis by arresting MYC-induced apoptosis, primarily through suppressing BIM. In *Eμ-myc* mice, homozygous *mnt* deletion decreased lymphoma incidence by enhancing apoptosis and reducing premalignant B-cells [67]. Other transcriptional regulators have been described to antagonize or cooperate with proto-oncoprotein MYC. For example, a tumor suppressor PRDM11 has been shown to interact in vivo with MYC during lymphomagenesis. Loss of *Prdm11* accelerates MYC-driven lymphomagenesis, regulating target genes in DLBCL cells such as *FOS* and *JUN.* Furthermore, patients with PRDM11-deficient DLBCLs have poorer survival [68]. A well-known transcription factor family, FOXO (Forkhead Box O) can also promote lymphomagenesis. Taking advantage of *vavP-MYC10* and *Eμ-myc* mice, loss of FoxO3 variant induced significant acceleration of tumorigenesis. Loss of FoxO3 increased myeloid cells on the blood, spleen and, bone marrow of *Eμ-myc* mice, as in *MYC10* mice. In vitro assays demonstrated that loss of FoxO3 had no impact on the cycling of preneoplastic and neoplastic *Eμ-myc* pre-B-cells, as well as, the selective pressure for mutation of the p53 pathway during lymphomagenesis was not altered [69]. Recent studies focused on FOXO1 protein [70], the highest FOXO protein expressed in lymphoid cells [71]. It was shown the abundant nuclear localization of FOXO1 in Burkitt’s lymphoma (BL). Using genome editing in human and mouse MYC-driven lymphoma, the study demonstrates its pro-proliferative and anti-apoptotic activity in BL and identify its nuclear localization as an oncogenic event in germinal center B-cell derived lymphomagenesis [70]. In B-cell lymphomas, *MYC* gene can be inserted in various positions of the IgH locus. Once translocated and having lost its normal control, MYC is constitutively expressed throughout the cell cycle in B-cells. Two major IgH transcriptional enhancers have been reported so far to be linked with the oncogene MYC as the cause of B-cell malignancies. The Eμ enhancer controls early events in B-cell maturation such as VDJ recombination [72,73] while the 3′ regulatory region (3′RR) controls late events in B-cell maturation such as IgH transcription [74], somatic hypermutation [75], and class switch recombination [76,77]. More recent reports have demonstrated that there are IgH intrachromosomal interactions between these 2 potent *cis*-acting enhancer elements that contribute to MYC deregulation. By transgenic and knock-in animal models that bring the oncogene *myc* under Eμ/3′RR transcriptional control, it was shown the essential contribution of both Eμ and 3′RR in B-cell lymphomagenesis. Of note, transgenic mice had a high rate of lymphoma emergence, increased proliferation, and the highest transcriptomic similarities to human BL [78].

Sabò et al. analyzed the genomic distribution of MYC during B-cell lymphomagenesis in the *Eμ-myc* transgenic mice and generated ChIP-seq profiles. Among other genes, they observed that MYC associated with regulatory elements of the *Ibtk* gene. Intriguingly, MYC binding intensity of *Ibtk* promoter increased during lymphoma progression [79]. *Ibtk* is a gene coding for a protein belonging to the Cul3-dependent ubiquitin ligase (CRL3^IBTK^) complex [80,81] and, it has been shown to act mainly by counteracting B-cells apoptosis during B-tumorigenesis [82], especially during MYC-driven lymphomagenesis [83]. Of note, loss of *Ibtk* delays the tumor onset and improves animal survival, by reducing the number of pre-cancerous B-cells of bone marrow and spleen. It is due principally to impaired viability and increased apoptosis of pre-cancerous B-cells. Consequently, it was observed the altered expression of proteins leading to apoptotic pathways dependent on MYC overexpression in pre-cancerous *Eμ-myc* mice, such as p53 and MCL-1 protein. Furthermore, a recent report on *Ibtk* shows that its haploinsufficiency also alters tumor microenvironment by enhancing MYC-driven vascularization in B-cell lymphomas [33].

Recent papers show that lncRNAs are pivotal in MYC-driven B-lymphoproliferative disorders. An example is lncRNA NEAT1 (Nuclear Paraspeckle Assembly Transcript 1). The aberrant expression of lncRNA NEAT1 is a common feature of DLBCL [84] and chronic myeloid leukemia (CML) [85]. In the latter, it was shown that MYC can bind and suppress NEAT1 expression, enhancing the imatinib-induced apoptosis [85]. MYC regulates cell proliferation of DLBCL via the NEAT1-miR-34b-5p-GLI1 signaling axis modulating DLBCL progression. A direct bound to the NEAT1 promoter allows MYC to regulate the NEAT1 level. In particular, knockdown of NEAT1 constrained cell proliferation and facilitated apoptosis of DLBCL cells through the miR-34b-5p-GLI1 pathway [86].

## 6. Regulatory Feedback Action of miRs during MYC-Driven Lymphomagenesis

Additionally, key microRNAs (miRs) (such as miR-34a and miR17-92) are related to MYC activation and play a vital role in some B-cell lymphomas [51]. The relevance of MYC-miRs circuits in B-cell functions is a novel insight into lymphomagenesis. MiRs interact with MYC acting as regulatory feedback that can influence the pre- or post-transcriptional expression of multiple genes, as shown in Table 2 and Figure 1. MiR-34a is reported to have tumor-suppressive activity in the oncogenesis of lymphoma and progression, contributing to p53-dependent apoptosis [87,88]. In DLBCL cells MYC mediated the repression of miR-34a resulting in a high proliferation of B-cell lymphoma by dysregulation of its target FOXP1 [89]. miR-34a and let7a are also targets of MYC repression establishing a feedback loop that aggravates and perpetuates the effects of MYC overexpression and may thus contribute to cancer progression [90]. MYC can indirectly repress several miRs by the recruitment of histone deacetylase (HDAC). It is known that the expression of miR-15a/miR-16-1 promotes apoptosis of cancer cells and suppresses tumorigenicity. The miR-15a/16-1 cluster directly down-regulates the anti-apoptotic protein BCL-2, MCL-1, CCND1, and WNT3A [91] and leads to induction of G0G1 arrest in tumor cells by suppressing cell cycle-positive regulators including CDK6 (Cyclin Dependent Kinase 6), Cyclins D and E [92]. Through epigenetic regulation and recruiting histone deacetylase 3 (HDAC3), MYC represses miR-15a/miR-16-1 expression in mantle cell and other non-Hodgkin B-cell lymphomas [93]. Indeed, MYC and EZH2 act in concert to silence tumor suppressor miRs in aggressive lymphoma cells. Of note, MYC recruited EZH2 to miR-26a promoter repressing miR-26a expression in aggressive lymphoma cell lines as well as primary lymphoma cells [94]. Concomitantly inhibitory action of both EZH2 and HDAC3 promoted transcriptional and epigenetic MYC-mediated repression of miR-29, resulting in down-regulation of miR-29 target pro-apoptotic genes and lymphoma growth suppression in vitro and in vivo [95]. MYC can work not only indirectly on the regulation of miRs but also as direct activator. The best example is the case of the oncogenic miR17-92 cluster. The miR17-92 polycistron at 13q31.3 is commonly amplified in several subtypes of aggressive lymphomas, including diffuse large B-cell lymphoma (DLBCL), mantle cell lymphoma (MCL), BL [96,97]. Several oncogenic transcription factors (TFs) regulate the expression of miR-17-92 cluster thus influencing its oncogenic activity. MYC was the first identified transcription regulator of miR-17-92 by directly binding to its genomic locus. The first model of MYC-miR-17-92 interaction was demonstrated in *Eμ-miR-17~92* transgenic mice, where the overexpression of miR-17-19b promotes the oncogenesis of MYC expressing B-cell [98]. MYC may suppress the expression of the pro-apoptotic *BIM* gene, a target of miR-17-92 thus explaining its tumor-promoting function, so contributing to maintaining survival and self-renewal [5]. Furthermore, the induction of the miR-17-92 cluster by MYC attenuates E2F1 protein expression, such that interruption of this regulatory loop results in DNA replication stress thus promoting lymphomagenesis [99]. The high miR-17-92 expression has been found in BL, confirming that the activation of the MYC/miR-17-92 axis is a general feature of this disease [100]. Of note, the typical translocation of MYC into the immunoglobulin heavy chain locus is observed in about 80% of BLs [101] and this overexpression can induce higher miR-17-92 levels. Further studies are needed to investigate the role of miR-17-92 in DLBCL, where the pro-apoptotic PTEN tumor suppressor gene could be involved as miR-19a target [102]. As it is also a direct MYC target, MYC-induced miR may coordinate the balance of cell proliferation and cell death in these lymphomas. On the other hand, previous studies have suggested PTEN as an indirect regulator of MYC activity through activation of the PI3K-AKT-GSK3β pathway. Down-regulation of the PTEN protein may represent yet a pathway of post-transcriptional MYC activation [103].

Furthermore, MYC acts as a negative regulator of *miR-144/451* expression by directly binding to its promoter region and, on its turn, is negatively regulated by miR-451. The expression of *miR-144/451* is commonly down-regulated in B-lymphomas, and therefore, the axis MYC -miR-144/451generates a positive feedback loop which sustains the persistently elevated high MYC levels, in B-lymphocytes. By using a mouse model knocked out for *miR-144/451* gene (*miR-144/451^−/−^*), it has been shown that the loss of *miR-144/451* is important to initiate and promote B-lymphomagenesis through the subsequent activation of MYC [104]. Collectively, these observations highlight the broad impact of MYC-mediated miRNA reprogramming on cellular survival and proliferation pathways, through controlling apoptosis.

## 7. Conclusions and Remarks

Compromised apoptotic signaling promote tumorigenesis, particularly together with oncogenic lesions that drive excess cell proliferation [105,106]. *MYC* gene alterations have been identified in B-cell neoplasms, and are usually associated with aggressive clinical behavior. Development of novel techniques in the field of immunology and new animal models have greatly accelerated our understanding of oncogenic mechanisms in these MYC-associated lymphomas. Mouse models are a powerful tool to reveal multiple genes implicated in the genesis of lymphoma and are extensively used to clarify the molecular mechanism of lymphoma, validating the gene function. MYC is a transcription factor that regulates several relevant biological cellular events. Here, we have highlighted the transcriptional regulation network of MYC during lymphomagenesis, the molecular mechanisms of MYC collaborations that lead to escape from apoptosis, and the circuits between MYC and miRs involved in the pathogenesis of B-cell lymphomas harboring MYC alterations. Moreover, from the examples discussed above, the scientific evidence is increasing on novel interactors of MYC. These cooperations are at the head of a cascade that ultimately triggers apoptosis pathways. The dynamic interactions between anti- and pro-apoptotic proteins and their regulation by oncogene MYC depicted in Figure 1, have uncovered novel opportunities for therapeutic intervention. Therefore, new agents designed to specifically target MYC and its interactors will need to be improved. MYC-driven pathways are extremely intricate, involving a huge number of interactors, and affecting several cellular processes such as cell survival, proliferation, differentiation, and apoptosis, sometimes conflicting with each other. A deeper understanding of the molecular mechanisms of MYC roles and its interactors in each B-cell lymphoma types provides a wide set of candidates for the development of a better personalized targeted therapy that in near future could lead to successful treatments with less toxicity.

## Figures and Tables

**Table 1:** Interactors involved in the transcriptional regulation of B-cell lymphomagenesis.

Interactor	Lymphoma Subtypes and Mouse Models	Targets of Interaction	Impact on Lymphomagenesis	References
lncRNA NEAT1	DLBCL, CML	miR-34b-5p-GLI1 pathway	Reduction	[84,85]
PRDM11	DLBCL	*FOS* and *JUN*	Reduction	[68]
MNT	*vavP-MYC10* and *Eμ-myc* mice	BIM	Acceleration	[2,66]
FOXO3/FOXO1	*vavP-MYC10* and *Eμ-myc* mice BL		Reduction	[69,70]
MAX	*Eμ-myc* mice		Reduction	[64]
IBTK	*Eμ-myc* mice	MCL-1 and p53	Acceleration	[67]
Eμ and 3′RR	c-myc-KIEμ, c-myc-KICμ, and c-myc-KICα mice BL-like lymphomas		Cooperate to induce	[78]

lncRNA: long non coding RNA; NEAT1: Nuclear Paraspeckle Assembly Transcript 1; PRDM11: PR/SET Domain 11; DLBCL: diffuse large B-cell lymphoma; CML: chronic myeloid lymphoma; GLI1: GLI Family Zinc Finger 1; MNT: MAX Network Transcriptional Repressor; FOXO3/FOX1: Forkhead Box O3/Forkhead Box O1; BL: Burkitt lymphoma; MAX: MYC Associated Factor X; IBTK: Inhibitor of Bruton Tyrosine Kinase.

**Table 2:** Major miRNAs involved in MYC-driven B-cell lymphomagenesis.

miRs	Functions	MYC Interactors	MYC Regulation	Lymphoma Subtypes	References
*miR-34a*	Tumor suppressor by promoting p53-dependent apoptosis	FOXP1	Negative regulation	DLBCL,FL,GC-DLBCL	[87,88,90]
*miR-15a/16-1* *cluster*	Tumor suppressor by targeting BCL2, Mcl-1, Cyclin D1 (miR-15a and miR 16-1); Tumor suppressor by inhibiting cell cycle-positive regulators including CDK6, Cyclins D and E (miR-16)	HDAC3	Negative regulation	MCL	[91,92,93]
*miR-26a*	Tumorsuppressor by promoting apoptosis	EZH2	Negative regulation	BL	[94]
*miR-29*	Tumor suppressorby targeting CDK6, Mcl-1, IGF-1R	HDAC3, EZH2	Negative regulation	MCL, GC-DLBCL, DLBCL, BL	[95]
*miR-17-92 cluster*	OncomiR by repressing the expression of BIM OncomiR by repressing the expression of PTEN (miR-19); OncomiR by down-regulating E2F1 (miR 17-20a)		Positive regulation	DLBCL, MCL, BL, GC-DLBCL	[96,97,98,99,100,101,102,103]
*miR144/451*	Positive feedback loop to safeguard the high level of MYC		Negative regulation	AML	[104]

miR: microRNA; DLBCL: diffuse large B-cell lymphoma; FL: follicular lymphoma; GC-DLBCL: germinal center-diffuse large B-cell lymphoma; FOXP1: Fork head Box P1; BCL2: B-cell lymphoma *2; Mcl-1:* Myeloid Cell Leukemia 1; MCL: mantle cell lymphoma; HDAC3: histone deacetylase 3; BL: Burkitt lymphoma; EZH2: Enhancer of zeste homolog 2; CDK6: Cyclin Dependent Kinase 6; IGF-1R: Insulin-like growth factor1 receptor; PTEN: Phosphatase and tensin homolog; AML: Acute Myeloid Leukemia.

## References

[1] Nesbit C.E., Tersak J.M., Prochownik E.V. (1999). MYC oncogenes and human neoplastic disease. Oncogene.

[2] Dang C.V., O’Donnell K.A., Zeller K.I., Nguyen T., Osthus R.C., Li F. (2006). The c-Myc target gene network. Semin. Cancer Biol..

[3] Eisenman R.N. (2001). Deconstructing myc. Genes Dev..

[4] Walz S., Lorenzin F., Morton J., Wiese K.E., Von Eyss B., Herold S., Rycak L., Dumay-Odelot H., Karim S., Bartkuhn M. (2014). Activation and repression by oncogenic MYC shape tumour-specific gene expression profiles. Nature.

[5] Li Y., Choi P.S., Casey S.C., Dill D.L., Felsher D.W. (2014). MYC through miR-17-92 suppresses specific target genes to maintain survival, autonomous proliferation, and a neoplastic state. Cancer Cell.

[6] Knoepfler P.S., Zhang X.Y., Cheng P.F., Gafken P.R., McMahon S.B., Eisenman R.N. (2006). Myc influences global chromatin structure. EMBO J..

[7] Orian A., Steensel B., Delrow J., Bussemaker H.J., Li L., Sawado T., Williams E., Loo L.W.M., Cowley S.M., Yost C. (2003). Genomic binding by the *Drosophila* myc, max, mad/mnt transcription factor network. Genes Dev..

[8] Malynn B.A., De Alboran I.M., O’Hagan R.C., Bronson R., Davidson L., DePinho R.A., Alt F.W. (2000). N-myc can functionally replace c-myc in murine development, cellular growth, and differentiation. Genes Dev..

[9] Cohen J.C., Scott D.K., Miller J., Zhang J., Zhou P., Larson J.E. (2004). Transient in utero knockout (TIUKO) of C-MYC affects late lung and intestinal development in the mouse. BMC Dev. Biol..

[10] Kelly K., Cochran B.H., Stiles C.D., Leder P. (1983). Cell-specific regulation of the c- myc gene by lymphocyte mitogens and platelet-derived growth factor. Cell.

[11] Sears R., Leone G., DeGregori J., Nevins J.R. (1999). Ras enhances Myc protein stability. Mol. Cell.

[12] Kress T.R., Sabo A., Amati B. (2015). MYC: Connecting selective transcriptional control to global RNA production. Nat. Rev. Cancer.

[13] Farrell A.S., Sears R.C. (2014). MYC degradation. Cold Spring Harb Perspect Med..

[14] Allen-Petersen B.L., Sears R.C. (2019). Mission Possible: Advances in MYC Therapeutic Targeting in Cancer. BioDrugs.

[15] Okuyama H., Endo H., Akashika T., Kato K., Inoue M. (2010). Downregulation of c-MYC protein levels contributes to cancer cell survival under dual deficiency of oxygen and glucose. Cancer Res..

[16] Dani C., Blanchard J.M., Piechaczyk M., El Sabouty S., Marty L., Jeanteur P. (1984). Extreme instability of myc mRNA in normal and transformed human cells. Proc. Natl. Acad. Sci USA.

[17] Wasylishen A.R., Penn L.Z. (2010). Myc: The beauty and the beast. Genes Cancer.

[18] Stine Z.E., Walton Z.E., Altman B.J., Hsieh A.L., Dang C.V. (2015). MYC, metabolism, and cancer. Cancer Discov..

[19] Karube K., Campo E. (2015). MYC alterations in diffuse large B-cell lymphomas. Semin. Hematol..

[20] Cai Q., Medeiros L.J., Xiaolu X., Yuong K.H. (2015). MYC-driven aggressive B-cell lymphomas: Biology, entity, differential diagnosis and clinical management. Oncotarget.

[21] Haralambieva E., Boerma E.-J., Van Imhoff G.W., Rosati S., Schuuring E., Müller-Hermelink H.K., Kluin P.M., Ott G. (2005). Clinical, immunophenotypic, and genetic analysis of adult lymphomas with morphologic features of Burkitt lymphoma. Am. J. Surg. Pathol..

[22] Niitsu N., Okamoto M., Miura I., Hirano M. (2009). Clinical features and prognosis of de novo diffuse large B-cell lymphoma with t (14; 18) and 8q24/c-MYC translocations. Leukemia.

[23] Valera A., Balagué O., Colomo L., Martínez A., Delabie J., Taddesse-Heath L., Jaffe E.S., Campo E. (2010). IG/MYC rearrangements are the main cytogenetic alteration in plasmablastic lymphomas. Am. J. Surg. Pathol..

[24] Wasylishen A.R., Chan-Seng-Yue M., Bros C., Dingar D., Tu W.B., Kalkat M., Chan P.-K., Mullen P.J., Huang L., Meyer N. (2013). MYC phosphorylation at novel regulatory regions suppresses transforming activity. Cancer Res..

[25] Kuttler F., Ame P., Clark H., Haughey C., Mougin C., Cahn J.Y., Dang C.V., Raffeld M., Fest T. (2001). C-myc box II mutations in Burkitt’s lymphoma-derived alleles reduce cell-transformation activity and lower response to broad apoptotic stimuli. Oncogenet.

[26] Herbst A., Hemann M.T., Tworkowski K.A., Salghetti S.E., Lowe S.W., Tansey W.P. (2005). A conserved element in Myc that negatively regulates its proapoptotic activity. EMBO Rep..

[27] Dalla-Favera R., Bregni M., Erikson J., Patterson D., Gallo R.C., Croce C.M. (1982). Human c-myc onc gene is located on the region of chromosome 8 that is translocated in Burkitt lymphoma cells. Proc. Natl. Acad. Sci. USA.

[28] Dejure F.R., Eilers M. (2017). MYC and tumor metabolism: Chicken and egg. EMBO J..

[29] Lefebure M., Tothill R.W., Kruse E., Hawkins E.D., Shortt J., Matthews G.M., Gregory G.P., Martin B.P., Kelly M.J., Todorovski I. (2017). Genomic characterisation of Emu-Myc mouse lymphomas identifies Bcor as a Myc co-operative tumour-suppressor gene. Nat. Commun..

[30] Greenwald R.J., Tumang J.R., Sinha A., Currier N., Cardiff R.D., Rothstein T.L., Faller D.V., Denis G.V.E. (2004). mu-BRD2 transgenic mice develop B-cell lymphoma and leukemia. Blood.

[31] Guo R., Jiang C., Zhang Y., Govande A., Trudeau S.J., Chen F., Fry C.J., Wolinsky E., Schineller M., Frost T.C. (2020). Myc controls the Epstein-Barr virus lytic switch. Mol. Cell..

[32] Poe J.C., Minard-Colin V., Kountikov E.I., Haas K.M., Tedder T.F. (2012). A c-Myc and surface CD19 signaling amplification loop promotes B cell lymphoma development and progression in mice. J. Immunol..

[33] Vecchio E., Fiume G., Mignogna C., Iaccino E., Mimmi S., Maisano D., Trapasso F., Quinto I. (2020). IBTK Haploinsufficiency Affects the Tumor Microenvironment of Myc-Driven Lymphoma in E-myc Mice. Int. J. Mol. Sci..

[34] Wahlstrom T., Henriksson M.A. (2015). Impact of MYC in regulation of tumor cell metabolism. Biochim. Biophys. Acta..

[35] Avagliano A., Fiume G., Pelagalli A., Sanità G., Ruocco M.S., Montagnani S., Arcucci A. (2020). Metabolic Plasticity of Melanoma Cells and Their Crosstalk With Tumor Microenvironment. Front. Oncol..

[36] Casey S.C., Baylot V., Felsher D.W. (2018). The MYC oncogene is a global regulator of the immune response. Blood.

[37] Atsaves V., Tsesmetzi N., Chioureas D., Kis L., Leventaki V., Drakos E., Panaretakis T., Grander D., Medeiros L.J., Young K.H. (2017). PD-L1 is commonly expressed and transcriptionally regulated by STAT3 and MYC in ALK-negative anaplastic large-cell lymphoma. Leukemia.

[38] Braun J., Felsher D.W., Goodglick L.A. (1992). c-myc, MHCI, and NK resistance in immunodeficiency lymphomas. Ann. NY Acad. Sci..

[39] Bisso A., Sabò A., Amati B. (2019). MYC in Germinal Center-derived lymphomas: Mechanisms and therapeutic opportunities. Immunol. Rev..

[40] Meyer N., Kim S.S., Penn L.Z. (2006). The Oscar-worthy role of Myc in apoptosis. Semin. Cancer Biol..

[41] Strasser A., Cory S., Adams J.M. (2011). Deciphering the rules of programmed cell death to improve therapy of cancer and other diseases. EMBO J..

[42] Dansen T.B., Whitfield J., Rostker F., Brown-Swigart L., Evan G.I. (2006). Specific requirement for Bax, not Bak, in Myc-induced apoptosis and tumor suppression in vivo. J. Biol. Chem..

[43] Eischen C.M., Woo D., Roussel M.F., Cleveland J.L. (2001). Apoptosis triggered by Myc-induced suppression of Bcl-XL or Bcl-2 is bypassed during lymphomagenesis. Mol. Cell. Biol..

[44] Juin P., Hunt A., Littlewood T., Griffiths B., Swigart L.B., Korsmeyer S., Evan G. (2002). c-Myc functionally cooperates with Bax to induce apoptosis. Mol. Cell. Biol..

[45] Tsujimoto Y., Yunis J., Onorato-Showe L., Erikson J., Nowell P.C., Croce C.M. (1984). Molecular cloning of the chromosomal breakpoint of B-cell lymphomas and leukemias with the t(11;14) chromosome translocation. Science.

[46] Vaux D.L., Cory S., Adams J.M. (1988). Bcl-2 gene promotes haemopoietic cell survival and cooperates with c-myc to immortalize pre-B cells. Nature.

[47] Dunleavy K. (2014). Double-hit lymphomas: Current paradigms and novel treatment approaches. Hematology Am. Soc. Hematol. Educ. Program..

[48] Adams C.M., Kim A.S., Mitra R., Choi J.K., Gong J.Z., Eischen C.M. (2017). BCL-W has a fundamental role in B cell survival and lymphomagenesis. J. Clin. Invest..

[49] Grabow S., Delbridge A.R., Aubrey B.J., Vandenberg C.J., Strasser A. (2016). Loss of a Single Mcl-1 Allele Inhibits MYC-Driven Lymphomagenesis by Sensitizing Pro-B Cells to Apoptosis. Cell. Rep..

[50] Grabow S., Kelly G.L., Delbridge A.R., Kelly P.N., Bouillet P., Adams J.M., Strasser A. (2016). Critical B-lymphoid cell intrinsic role of endogenous MCL-1 in c-MYC-induced lymphomagenesis. Cell. Death Dis..

[51] Yu L., Yu T.T., Young K.H. (2019). Cross-talk between Myc and p53 in B-cell lymphomas. Chronic Dis. Transl. Med..

[52] Zindy F., Eischen C.M., Randle D.H., Kamijo T., Cleveland J.L., Sherr C.J., Roussel M.F. (1998). Myc signaling via the ARF tumor suppressor regulates p53-dependent apoptosis and immortalization. Genes Dev..

[53] Finch A., Prescott J., Shchors K., Hunt A., Soucek L., Dansen T.B., Swigart L.B., Evan G.I. (2006). Bcl-xL gain of function and p19 ARF loss of function cooperate oncogenically with Myc in vivo by distinct mechanisms. Cancer Cell.

[54] Eischen C.M., Weber J.D., Roussel M.F., Sherr C.J., Cleveland J.L. (1999). Disruption of the ARF–Mdm2–p53 tumor suppressor pathway in Myc-induced lymphomagenesis. Genes Dev..

[55] Schmitt C.A., McCurrach M.E., De Stanchina E., Wallace-Brodeur R.R., Lowe S.W. (1999). INK4a/ARF mutations accelerate lymphomagenesis and promote chemoresistance by disabling p53. Genes Dev..

[56] Bouchard C., Lee S., Paulus-Hock V., Loddenkemper C., Eilers M., Schmitt C.A. (2007). FoxO transcription factors suppress Myc-driven lymphomagenesis via direct activation of Arf. Genes Dev..

[57] Alt J.R., Greiner T.C., Cleveland J.L., Eischen C.M. (2003). Mdm2 haplo-insufficiency profoundly inhibits Myc-induced lymphomagenesis. EMBO J..

[58] Jacobs J.J., Scheijen B., Voncken J.W., Kieboom K., Berns A., Van Lohuizen M. (1999). Bmi-1 collaborates with c-Myc in tumorigenesis by inhibiting c-Myc-induced apoptosis via INK4a/ARF. Genes Dev..

[59] Dickins R.A., Hemann M.T., Zilfou J.T., Simpson D.R., Ibarra I., Hannon G.J., Lowe S.W. (2005). Probing tumor phenotypes using stable and regulated synthetic microRNA precursors. Nature Genet..

[60] Haupt Y., Bath M.L., Harris A.W., Adams J. (1993). Bmi-1 trans-gene induces lymphomas and collaborates with myc in tumourigenesis. Oncogenet.

[61] Garrison S.P., Jeffers J.R., Yang C., Nilsson J.A., Hall M.A., Rehg J.E., Yue W., Yu J., Zhang L., Onciu M. (2008). Selection against PUMA gene expression in Myc-driven B-cell lymphomagenesis. Mol. Cell. Biol..

[62] Willis S.N., Fletcher J.I., Kaufmann T., Van Delft M.F., Chen L., Czabotar P.E., Ierino H., Lee E.F., Fairlie W.D., Bouillet P. (2007). Apoptosis initiated when BH3 ligands engage multiple Bcl-2 homologs, not Bax or Bak. Science.

[63] Blackwood E.M., Eisenman R.N. (1991). Max: A helix-loop-helix zipper protein that forms a sequence-specific DNA-binding complex with Myc. Science.

[64] Mathsyaraja H., Freie B., Cheng P.F., Babaeva E., Catchpole J.T., Janssens D., Henikoff S., Eisenman R.N. (2019). *Max* deletion destabilizes MYC protein and abrogates *Eµ-Myc* lymphomagenesis. Genes Dev..

[65] Yang G., Hurlin P.J. (2017). MNT and emerging concepts of MNT-MYC antagonism. Genes.

[66] Campbell K.J., Vandenberg C.J., Anstee N.S., Hurlin P.J., Cory S. (2017). Mnt modulates Myc-driven lymphomagenesis. Cell Death Differ..

[67] Nguyen H.V., Vandenberg C.J., Ng A.P., Robati M.R., Anstee N.S., Rimes J., Hawkins E.D., Cory S. (2020). Development and survival of MYC-driven lymphomas require the MYC antagonist MNT to curb MYC-induced apoptosis. Blood.

[68] Fog C.K., Asmar F., Côme C., Jensen K.T., Johansen J.V., Kheir T.B., Jacobsen L., Friis C., Louw A., Rosgaard L. (2015). Loss of PRDM11 promotes MYC-driven lymphomagenesis. Blood.

[69] Vandenberg C.J., Motoyama N., Cory S. (2016). FoxO3 suppresses Myc-driven lymphomagenesis. Cell Death Dis..

[70] Kabrani E., Chu V.T., Tasouri E., Sommermann T., Baßler K., Ulas T., Zenz T., Bullinger L., Schultze J.L., Rajewsky K. (2018). Nuclear FOXO1 promotes lymphomagenesis in germinal center B cells. Blood.

[71] Dejean A.S., Hedrick S.M., Kerdiles Y.M. (2011). Highly specialized role of Forkhead box O transcription factors in the immune system. Antioxid Redox Signal..

[72] Perlot T., Alt F.W., Bassing C.H., Suh H., Pinaud E. (2005). Elucidation of IgH intronic enhancer functions via germ-line deletion. Proc Natl Acad Sci USA.

[73] Marquet M., Garot A., Bender S., Carrion C., Rouaud P., Lecardeur S., Denizot Y., Cogné M., Pinaud E. (2014). The Em enhancer region influences H chain expression and B cell fate without impacting IgVH repertoire and immune response in vivo. J. Immunol..

[74] Saintamand A., Rouaud P., Garot A., Rios G., Cogné M., Denizot Y. (2015). The IgH 39 regulatory region governs m chain transcription in mature B lymphocytes and the B cell fate. Oncotarget.

[75] Rouaud P., Vincent-Fabert C., Saintamand A., Fiancette R., Marquet M., Robert I., Reina-San-Martin B., Pinaud E., Cogné M. (2013). The IgH 39 regulatory region controls somatic hypermutation in germinal center B cells. J. Exp. Med..

[76] Saintamand A., Rouaud P., Saad F., Rios G., Cogne Ã.Å.M., Denizot Y. (2015). Elucidation of IgH 39 region regulatory role during class switch recombination via germline deletion. Nat. Commun..

[77] Rouaud P., Saintamand A., Saad F., Carrion C., Lecardeur S., Cogné M., Denizot Y. (2014). Elucidation of the enigmatic IgD class-switch recombination via germline deletion of the IgH 39 regulatory region. J. Exp. Med..

[78] Ghazzaui N., Issaoui H., Ferrad M., Carrion C., Cook-Moreau J., Denizot Y., Boyer F. (2020). Eμ and 3’RR transcriptional enhancers of the IgH locus cooperate to promote c-myc-induced mature B-cell lymphomas. Blood Adv..

[79] Sabo A., Kress T.R., Pelizzola M., De Pretis S., Gorski M.M., Tesi A., Morelli M.J., Bora P., Doni M., Verrecchia A. (2014). Selective transcriptional regulation by Myc in cellular growth control and lymphomagenesis. Nature.

[80] Pisano A., Ceglia S., Palmieri C., Vecchio E., Fiume G., De Laurentiis A., Mimmi S., Falcone C., Iaccino E., Scialdone A. (2015). CRL3IBTK regulates the tumor suppressor Pdcd4 through ubiquitylation coupled to proteasomal degradation. J. Biol. Chem..

[81] Fiume G., Scialdone A., Rizzo F., De Filippo M.R., Laudanna C., Albano F., Golino G., Vecchio E., Pontoriero M., Mimmi S. (2016). IBTK Differently Modulates Gene Expression and RNA Splicing in HeLa and K562 Cells. Int. J. Mol. Sci..

[82] Albano F., Chiurazzi F., Mimmi S., Vecchio E., Pastore A., Cimmino C., Frieri C., Iaccino E., Pisano A., Golino G. (2018). The expression of inhibitor of bruton’s tyrosine kinase gene is progressively up regulated in the clinical course of chronic lymphocytic leukaemia conferring resistance to apoptosis. Cell Death Dis..

[83] Vecchio E., Golino G., Pisano A., Albano F., Falcone C., Ceglia S., Iaccino E., Mimmi S., Fiume G., Giurato G. (2019). IBTK contributes to B-cell lymphomagenesis in Eμ-myc transgenic mice conferring resistance to apoptosis. Cell Death Dis..

[84] Deng L., Jiang L., Tseng K.F., Liu Y., Zhang X., Dong R., Zhigangc L., Xiujua W. (2018). Aberrant NEAT1_1 expression may be a predictive marker of poor prognosis in diffuse large B cell lymphoma. Cancer Biomark..

[85] Zeng C., Liu S., Lu S., Yu X., Lai J., Wu Y., Chen S., Wang L., Yu Z., Luo G. (2018). The c-Myc-regulated lncRNA NEAT1 and paraspeckles modulate imatinib-induced apoptosis in CML cells. Mol. Cancer.

[86] Qian C.S., Li L.J., Huang H.W., Yang H.F., Wu D.P. (2020). MYC-regulated lncRNA NEAT1 promotes B cell proliferation and lymphomagenesis via the miR-34b-5p-GLI1 pathway in diffuse large B-cell lymphoma. Cancer Cell. Int..

[87] Chang T.C., Wentzel E.A., Kent O.A., Ramachandran K., Mullendore M., Lee K.H., Feldmann G., Yamakuchi M., Ferlito M., Lowenstein C.J. (2007). Transactivation of miR-34a by p53 broadly influences gene expression and promotes apoptosis. Mol. Cell.

[88] Raver-Shapira N., Marciano E., Meiri E., Spector Y., Rosenfeld N., Moskovits N., Bentwich Z., Oren M. (2007). Transcriptional activation of miR-34a contributes to p53-mediated apoptosis. Mol. Cell.

[89] Craig V.J., Cogliatti S.B., Imig J., Renner C., Neuenschwander S., Rehrauer H., Schlapbach R., Dirnhofer S., Tzankov A., Müller A. (2011). Myc-mediated repression of microRNA-34a promotes high-grade transformation of B-cell lymphoma by dysregulation of FoxP1. Blood.

[90] Christoffersen N.R., Shalgi R., Frankel L.B., Leucci E., Lees M., Klausen M., Pilpel Y., Nielsen F.C., Oren M., Lund A.H. (2010). p53-independent upregulation of miR-34a during oncogene-induced senescence represses MYC. Cell Death Differ..

[91] Aqeilan R.I., Calin G.A., Croce C.M. (2010). miR-15a and miR-16-1 in cancer: Discovery, function and future perspectives. Cell Death Differ..

[92] Linsley P.S., Schelter J., Burchard J., Kibukawa M., Martin M.M., Bartz S.R., Johnson J.M., Cummins J.M., Raymond C.K., Dai H. (2007). Transcripts targeted by the microRNA-16 family cooperatively regulate cell cycle progression. Mol. Cell Biol..

[93] Zhang X., Chen X., Lin J., Lwin T., Wright G., Moscinski L.C., Dalton W.S., Seto E., Wright K., Sotomayor E. (2012). Myc represses miR-15a/miR-16-1 expression through recruitment of HDAC3 in mantle cell and other non-Hodgkin B-cell lymphomas. Oncogene.

[94] Zhao X., Lwin T., Zhang X., Huang A., Wang J., Marquez V.E., Chen-Kiang S., Dalton W.S., Sotomayor E., Tao J. (2013). Disruption of the MYC-miRNA-EZH2 loop to suppress aggressive B-cell lymphoma survival and clonogenicity. Leukemia.

[95] Zhang X., Zhao X., Fiskus W., Lin J., Lwin T., Rao R., Zhang Y., Chan J.C., Fu K., Marquez V.E. (2012). Coordinated silencing of MYC-mediated miR-29 by HDAC3 and EZH2 as a therapeutic target of histone modification in aggressive B-Cell lymphomas. Cancer Cell.

[96] Lenz G., Wright G.W., Emre N.C., Kohlhammer H., Dave S.S., Davis R.E., Carty S., La L.T., Shaffer A.L., Xiao W. (2008). Molecular subtypes of diffuse large B-cell lymphoma arise by distinct genetic pathways. Proc. Natl. Acad. Sci. USA.

[97] Dal Bo M., Bomben R., Hernández L., Gattei V. (2015). The MYC/miR-17-92 axis in lymphoproliferative disorders: A common pathway with therapeutic potential. Oncotarget. Review.

[98] Sandhu S.K., Fassan M., Volinia S., Lovat F., Balatti V., Pekarsky Y., Croce C.M. (2013). B-cell malignancies in microRNA Emu-miR-17~92 transgenic mice. Proc. Natl. Acad. Sci. USA.

[99] Oduor C.I., Kaymaz Y., Chelimo K., Otieno J.A., Ong’echa J.M., Moormann A.M., Bailey J.A. (2017). Integrative microRNA and mRNA deep-sequencing expression profiling in endemic Burkitt lymphoma. BMC Cancer.

[100] Schmitz R., Young R.M., Ceribelli M., Jhavar S., Xiao W., Zhang M., Wright G., Shaffer A.L., Hodson D.J., Buras E. (2012). Burkitt lymphoma pathogenesis and therapeutic targets from structural and functional genomics. Nature.

[101] Boxer L.M., Dang C.V. (2001). Translocations involving c-myc and c-myc function. Oncogene.

[102] Olive V., Bennett M.J., Walker J.C., Ma C., Jiang I., Cordon-Cardo C., Li Q.J., Lowe S.W., Hannon G.J., He L. (2009). miR-19 is a key oncogenic component of mir-17-92. Genes Dev..

[103] Bonnet M., Loosveld M., Montpellier B., Navarro J.M., Quilichini B., Picard C., DiCristofaro J., Bagnis C., Fossat C., Hernandez L. (2011). Posttranscriptional deregulation of MYC via PTEN constitutes a major alternative pathway of MYC activation in T-cell acute lymphoblastic leukemia. Blood.

[104] Ding L., Zhang Y., Han L., Fu L., Mei X., Wang J., Itkow J., Elabid A.E.I., Pang L., Yu D. (2018). Activating and sustaining c-Myc by depletion of miR-144/451 gene locus contributes to B-lymphomagenesis. Oncogene.

[105] Pontoriero M., Fiume G., Vecchio E., De Laurentiis A., Albano F., Iaccino E., Mimmi S., Pisano A., Agosti V., Giovannone E. (2019). Activation of NF-kB in B cell receptor signaling through Bruton’s tyrosine kinase-dependent phosphorylation of IκB-α. J. Mol. Med..

[106] Hotchkiss R.S., Strasser A., McDunn J.E., Swanson P.E. (2009). Cell death. N. Engl. J. Med..

